# Expression level of chromodomain Y (CDY): potential marker for prediction of sperm recovery in non-obstructive azoospermia 

**Published:** 2016-06

**Authors:** Neda Heydarian, Raha Favaedi, Mohammad Ali Sadighi Gilani, Maryam Shahhoseini

**Affiliations:** 1 *Department of Biology, Faculty of Science, Science and Research Branch, Islamic Azad University, Tehran, Iran.*; 2 *Department of Genetics, Reproductive Biomedicine Research Center, Royan Institute for Reproductive Biomedicine, ACECR, Tehran, Iran.*; 3 *Department of Andrology, Reproductive Biomedicine Research Center, Royan Institute for Reproductive Biomedicine, ACECR, Tehran, Iran.*; 4 *Department of Urology, Shariati Hospital, Tehran University of Medical Sciences, Tehran, Iran.*

**Keywords:** *Azoospermia*, *CDY1*, *Gene expression*, *Marker*

## Abstract

**Background:**

The availability of testis specific genes will be of help in choosing the most promising biomarkers for the detection of testicular sperm retrieval in patients with non-obstructive azoospermia (NOA). Testis specific chromodomain protein Y 1 (CDY1) is a histone acetyltransferase which concentrates in the round spermatid nucleus, where histone hyperacetylation occurs and causes the replacement of histones by the sperm-specific DNA packaging proteins, TNPs and PRMs.

**Objective:**

The aim was to evaluate *CDY1* gene as a marker for predicting of successful sperm retrieval in NOA patients.

**Materials and Methods:**

This research was conducted on 29 patients with NOA who had undergone testicular sperm extraction (TESE) procedure. NOA patients were subdivided into patients with successful sperm retrieval (NOA+, n=12) and patients with unsuccessful sperm retrieval (NOA-, n=17). Relative expression of *CDY1* gene and chromatin incorporation of CDY1 protein were measured by quantitative real-time polymerase chain reaction (qRT-PCR) and ELISA assay, respectively.

**Results:**

Quantification of mRNA relative expression and incorporation of CDY1 protein in chromatin showed significant lower expressions and protein levels of CDY1 in testis tissues of NOA- in comparison to NOA+ group.

**Conclusion:**

The findings in this study demonstrated a correlation between the low levels of CDY1 function and unsuccessful sperm recovery in the testicular tissues of NOA- compared to NOA+ patients. Therefore, it can be reasonable to consider *CDY1* as a potential biomarker for predicting the presence of spermatozoa, although the claim needs more samples to be confirmed.

## Introduction

Non-obstructive azoospermia (NOA) affects 10% of infertile men and is distinguished in 60% of azoospermic men (1). NOA characterizes the condition in which there are no sperm in semen due to defect in spermatogenesis in the testes, rather than due to post testicular obstruction of the excurrent ducts (2). If sperm can be retrieved by testicular sperm extraction (TESE), Men with NOA may be able to fertilize by intracytoplasmic sperm injection (ICSI) (3, 4).

However, it has been reported that about 50% of NOA patients had unnecessary surgeries (4). It would be helpful to be able to identify a spermatogenesis molecular marker that exists when full spermatogenesis is achieved and can predicts successful sperm retrieval in patients with NOA undergoing TESE (5).

Spermatogenesis occurs by successive mitotic, meiotic, and post meiotic processes. Genes that are expressed during spermatogenesis encode necessary proteins for specific stages as well as for holding the general housekeeping functions of the cells involved (6). During spermatogenesis, correct histone-to-protamine exchange is known to cause chromatin condensation and is essential for the complete differentiation of spermatids (7). This histone-to-protamine exchange is facilitated by a wave of core histone acetylation during spermiogenesis, one of the best described changes in histone post-translational modifications. Testis specific chromo domain protein1, named CDY1 is a histone acetyltransferase which associates with up regulation of post meiotic genes of spermatogenesis such as transition proteins (*TNPs)* and protamins (*P*RMs) (8). Previous studies have shown that the expression of *CDY1* in testicular tissue correlates with complete spermatogenesis (6, 9, 10, 12). 

Several lines of studies have been conducted to better understand the role of *CDY* genes in the spermatogenesis process (11-13). Kleiman *et al* confirmed the usefulness of analyzing the *CDY1* transcripts as effective candidates for predicting the presence of complete spermatogenesis (14). A recent study showed that deletion of *CDY1 *gene is a significant risk factor for male infertility independent of sperm concentration (15). 

Our objective was to evaluate mRNA expression and chromatin incorporation of CDY1 by quantitative real-time polymerase chain reaction (qRT-PCR) and chromatin ELISA technique, in NOA patients who underwent TESE. We performed the assessment in an effort to suggest *CDY1* expression analysis as a diagnostic test to predict the presence of retrievable testicular sperm in NOA patients who need to repeat TESE procedure.

## Materials and methods

In this case-control study, 29 testicular biopsy specimens were collected from NOA patients referred to ROYAN Institute to underwent sperm extraction from 2012 to 2013 (Table I). All testicular samples were obtained from TESE procedures performed in an attempt to obtain sperm for ICSI. The present study was approved by the Institutional Review Board of the Royan Institute and written informed consent was given by all patients, permitting the use of their tissue samples in this study.

TESE surgery was performed first on one testis. In cases that sperm retrieval was failed from one testis, searching for spermatozoa was performed on both testes. All testicular tissue evaluations were meticulously performed from the same location in the testis, based on the nonhomogeneous nature of this tissue. The absence of sperm in TESE- samples may not be a conclusive mark of the sperm absence in testis due to the heterogeneous nature of testis specimens, but as a whole and with a high probability these testicular samples are lack of sperm. 

Through histopathologic assessment, these samples distributed into 4 groups: hypo spermatogenesis in 7 patients, incomplete maturation arrest in 3 patients, complete maturation arrest in 11 patients, and Sertoli cell-only syndrome in 8 patients. These patients were classiﬁed into two groups based on their sperm extraction results: patients with successful sperm retrieval (NOA+, n=12) as control and patients with unsuccessful sperm retrieval (NOA-, n=17) as case group (Table II).

**RNA isolation and cDNA synthesis**

Total RNA was extracted from the tissue samples using Trizol reagent (Invitrogen, cat#. 15596-018) according to the manufacturer's protocol. Then the tissue lysate was treated with DNase-I (TAKARA, cat#. K301BA) to exclude DNA contamination. Purity and concentration of RNA samples were determined using Nanodrop 2000 spectrophotometer (Thermo Scientific), then cDNA synthesis was performed using RevertAid First strand cDNA Synthesis Kit (Fermentas, cat#. K1632). Produced cDNA were finally used as templates for detection of *CDY1 *expression.

**Quantitative real-time PCR (qRT-PCR)**

The cDNA samples were subjected to qRT-PCR using SYBR Green PCR master mix (Applied Biosystems) on a Step One plus Real-Time PCR System (Applied Biosystem) and with designed primers listed in table III. The amplification profile was as follows: initial denaturation at 95^o^C for 4 min, followed by 40 cycles of denaturation at 95^o^C for 10 sec, annealing at 60^o^C for 30 sec, extension at 72^o^C for 30 sec, and a final extension at 72^o^C for 10 min. Samples were normalized with the expression of β-actin as internal control. The relative gene expression was calculated by using 2^-ΔΔCt^ quantitative method, in which the parameter, Ct (threshold cycle), indicates the fractional cycle number where the fluorescent signal reaches detection threshold.

**Sheared chromatin preparation**

After homogenization of testis samples in cold PBS, chromatin of each tissue sample was fixed by 1% final concentration (w/v) of formaldehyde then shacked gently on a platform at room temperature for 10 min. In next step glycine was added to homogenized tissue to reach final concentration of 125 mM due to quench the reaction of formaldehyde. After washing tissue pellet with cold PBS, lysis buffer was added and then pellet was sonicated for 10 min (30" on/30" off, UCD200- Bioruptor sonication system, Diagenode) to get soluble sheared chromatin. After shearing, chromatin was centrifuged at 14000 gr for 5 min to remove cellular debris.

**Enzyme-linked immunoabsorbent assay (ELISA)**

In order to compare the total amount of CDY1 protein in sheared chromatin, CDY1 protein levels were evaluated by ELISA technique using anti-CDY1 antibody (Sigma, cat#. AV48645) in testicular tissue of all the studied patients according to Dai and Rasmussen (16). Optical absorption was read at 450 nm on an ELISA-reader, Thermo Scientific (MULTISKAN Spectrum).

**Statistical analysis**

Quantitative variables were expressed as mean±SEM. Statistical comparisons were determined by independent samples T-test. Differences were considered statistically significant at least at p≤0.05. Statistical analysis was performed using SPSS for windows (version 20).

## Results

**Clinical characteristics of infertile men**

There were no significant differences in any clinical parameters including: age, testicular volumes or level of FSH, LH and testosterone between the successful and unsuccessful TESE groups (NOA+ and NOA- respectively) (Table I).

**Expression analysis of** ***CDY1*** **gene**

**Figure 1:**
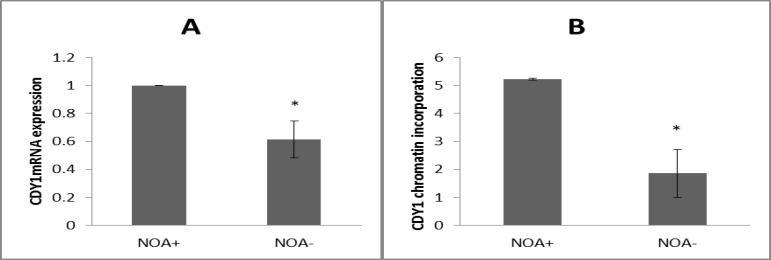
A) Gene expression and B) chromatin incorporation of CDY1 protein in NOA+ and NOA**-** groups. All data are presented as mean ± SEM and statistical comparisons were determined by independent samples T-test (NOA: non-obstructive azoospermia

*CDY1* gene expression was evaluated by quantitative real-time PCR analysis in testicular specimens classified into NOA+ and NOA- groups (Figure 1A). β-actin expression, which was confirmed in all 29 analyzed biopsies, was omitted from figure 1A. Quantification of mRNA relative expression showed a significant lower expression of *CDY1* gene in testis tissues of NOA- group in comparison to NOA+ group. The significance was calculated at p≤0.05 level.

**Chromatin incorporation of CDY1 protein in testicular biopsies**

Total protein levels of CDY1 into chromatin of the testis tissue sections were evaluated by ELISA using anti-CDY1 antibody. The quantitative data demonstrated a significant decrease of binded CDY1 protein in the chromatin fractions of specimens with NOA- in comparison to NOA+ (Figure 1B). The result was significant at p≤0.05 level.

**Table I:** The clinical characteristics of patients with NOA+ and NOA**-**groups

**Groups**	**NOA +**	**NOA-**
Number of patients	12	17
Age of patients (mean ± SD) a	34 ± 3.22	34.11 ± 2.97
Genetic analysis	46XY/ normal AZF	46XY/ normal AZF
Serum LH (mIU/ml) (mean ± SD) a	7.47 ± 1.94	7.14 ± 1.27
Serum FSH (mIU/ml) (mean ± SD) a	11.62 ± 3.55	12.83 ± 1.85
Serum testosterone(ng/ml)(mean ± SD) a	3.42 ± 0.82	4.92 ± 0.61

a No signiﬁcant difference, independent samples T-Test.

**Table II:** Number of specimens included in each group

**Groups **	**NOA +**	**NOA-**
Hypospermatogenesis	7	-
Incomplete maturation arrest	3	-
Complete maturation arrest	-	11
Sertoli cell-only syndrome	-	8

**Table III:** Real-time RT-PCR primers used in this study

**Genes **	**Primer sequences (5'→3')**	**Length(bp)**	**Tm (** **°C** **)**
*CDY1*	F: TTAATAGACCCATTAGCAGCCA R: ATTTCTTTGCCCACCTTTCAC	78	60
*ACTB*	F:AGCACAGAGCCTCGCCTT R:CACGATGGAGGGGAAGAC	163	60

## Discussion

Recent studies have emphasized on the need of more sensitive tools for diagnose spermatogenesis in the testes of azoospermic men (17). The assessment of patients with NOA routinely includes semen evaluation, hormone analysis, and cytogenetic testing, which can be enlarged to include genetic studies such as Y-microdeletions (17). As TESE and ICSI are invasive procedures for both man and the woman, identification of a parameter to predict the presence of sperm production in those patients would be of considerable value (18). Until serum markers become available for this purpose, evaluation of critical genes involved in the process of human spermatogenesis seems to be crucial for finding potential biomarkers (19). 

In the present study, we aimed to correlate the CDY1 expression to the successful testicular sperm retrieval in NOA patients. Accordingly, we investigated *CDY1* mRNA relative expression in testis tissue of NOA patients. Our findings revealed that CDY1 expression was significantly higher in patients with successful sperm retrieval than in patients with failed retrieval (NOA+ and NOA- groups respectively) (Figure 1A). To verify this possibility, incorporation of the protein into chromatin extracts of all the NOA patients was assessed using chromatin ELISA. The result also revealed a deficiency in CDY1 proteins by expression in human testes with NOA- compared with NOA+ group (Figure 1B). Concerning these findings, expression profile of CDY1 is considered to be associated with predicting successful sperm recovery. Likely, patients with high expression of CDY1 have more chance for successful sperm retrieval than patients with low expression of this testis specific histone modifier factor. These results indicate that the measurement of CDY1 transcript levels potentially may be useful in prediction of the presence of sperm in testis. 

Kleiman *et al* showed that, if expression detection of CDY1 was checked parallel with BOULE (a member of deleted in azoospermia DAZ gene family), the predictive power of the experiment was enhanced by having an additive efficacy (14). Our findings were in agreement with previous studies which have shown that expression of CDY1 in testicular tissue correlates with a complete spermatogenesis process and it can be considered as a predictive marker for successful testicular sperm retrieval (6, 12, 14).

If we fail to identify spermatozoa, despite detecting high expressions of transcript, it may be worthwhile to repeat TESE until the success of sperm retrieval. In other words, as testing *CDY1* gene expression is a cost effective test, it will spare some patients from undergoing unnecessary repeated surgical procedures. On the other hand, in our study we found no significant difference in clinical testicular sperm retrieval parameters including semen analysis, testicular volumes and level of FSH, LH and testosterone among NOA+ and NOA- patients. Bearing this in mind, the detection of CDY1 expression in the men confirms the previous findings to consider CDY1 as a potential biomarker for predicting the presence of spermatozoa in testicular tissue and may serve as a predictive test if repeated TESE is required. 

Further research with a large number of patients is indispensable for better confirming of the promising molecular marker in order to provide highly valuable information for complementing the histological analysis in predicting successful testicular sperm retrieval.

## Conclusion

We conducted the research to further confirm *CDY1* gene as a counseling biomarker for doctors treating azoospermia-related infertility and for patients to avoid undergoing unnecessary testicular sperm extraction. The expression profile and chromatin incorporation of CDY1 have measured based on a new categorizing of NOA patients: with successful and unsuccessful TESE procedure (TESE+ and TESE-, respectively). *CDY1* will contribute to discover if full spermatogenesis is occurred or not and helps infertile men for sperm retrieval, until serum markers are emerged.

## Note

***This article extracted from M.Sc. thesis. (Neda Heydarian)***
